# Dosage compensation and DNA methylation landscape of the X chromosome in mouse liver

**DOI:** 10.1038/s41598-018-28356-3

**Published:** 2018-07-04

**Authors:** Christopher G. Duncan, Sara A. Grimm, Daniel L. Morgan, Pierre R. Bushel, Brian D. Bennett, NISC Comparative Sequencing Program, Beatrice B. Barnabas, Gerard G. Bouffard, Shelise Y. Brooks, Holly Coleman, Lyudmila Dekhtyar, Xiaobin Guan, Joel Han, Shi-ling Ho, Richelle Legaspi, Quino L. Maduro, Catherine A. Masiello, Jennifer C. McDowell, Casandra Montemayor, James C. Mullikin, Morgan Park, Nancy L. Riebow, Karen Schandler, Brian Schmidt, Christina Sison, Raymond Smith, Sirintorn Stantripop, James W. Thomas, Pamela J. Thomas, Meghana Vemulapalli, Alice C. Young, John D. Roberts, Frederick L. Tyson, B. Alex Merrick, Paul A. Wade

**Affiliations:** 1 Epigenetics and Stem Cell Biology Laboratory, National Institute of Environmental Health Sciences, Research Triangle Park, NC, USA; 2 Integrative Bioinformatics, National Institute of Environmental Health Sciences, Research Triangle Park, NC USA; 3 Division of the National Toxicology Program, National Institute of Environmental Health Sciences, Research Triangle Park, NC USA; 4 Biostatistics and Computational Biology Branch, National Institute of Environmental Health Sciences, Research Triangle Park, NC USA; 5 Division of Extramural Research and Training, National Institute of Environmental Health Sciences, Research Triangle Park, NC USA; 6 NIH Intramural Sequencing Center, National Human Genome Research Institute, Rockville, MD USA

## Abstract

DNA methylation plays a key role in X-chromosome inactivation (XCI), a process that achieves dosage compensation for X-encoded gene products between mammalian female and male cells. However, differential sex chromosome dosage complicates genome-wide epigenomic assessments, and the X chromosome is frequently excluded from female-to-male comparative analyses. Using the X chromosome in the sexually dimorphic mouse liver as a model, we provide a general framework for comparing base-resolution DNA methylation patterns across samples that have different chromosome numbers and ask at a systematic level if predictions by historical analyses of X-linked DNA methylation hold true at a base-resolution chromosome-wide level. We demonstrate that sex-specific methylation patterns on the X chromosome largely reflect the effects of XCI. While our observations concur with longstanding observations of XCI at promoter-proximal CpG islands, we provide evidence that sex-specific DNA methylation differences are not limited to CpG island boundaries. Moreover, these data support a model in which maintenance of CpG islands in the inactive state does not require complete regional methylation. Further, we validate an intragenic non-CpG methylation signature in genes escaping XCI in mouse liver. Our analyses provide insight into underlying methylation patterns that should be considered when assessing sex differences in genome-wide methylation analyses.

## Introduction

Sexual dimorphism of gene expression is commonly observed in mammalian tissues, including liver, and results from genetic and hormonal factors^1^. Hormonal factors, predominantly sex-dependent patterns of pituitary growth hormone (GH) release, regulate the bulk of sex-biased gene expression in liver^2–4^. Likewise, phenotypic responses in mouse liver are confounded by inherent sex-dependent differential susceptibilities to hepatocarcinogenesis^5–7^. While X chromosome biology differs significantly between the sexes, the regulation of X-linked genes and their impacts on sexual dimorphism of the liver are less characterized.

Mammalian females have two X chromosomes, while males have one. Dosage compensation for X-linked genes between mammalian female (XX) and male (XY) individuals is achieved through transcriptional inactivation of one of the two X chromosomes in each female cell early in development^8^. Decades of research have shed light on the process of XCI, including its developmental regulation and the roles of noncoding RNAs and epigenetic mechanisms^9–12^. Compared to the active X chromosome (Xa), the inactive X chromosome (Xi) adopts a drastically different three-dimensional structural conformation as it becomes silent and heterochromatic^13–15^. Correspondingly, the Xi has long been associated with specific DNA methylation patterns, which play a key role in maintaining the inactive state^16–20^, while the Xa displays allele-specific methylation concentrated at gene bodies^21^.

Recent studies have characterized X-chromosome-wide CpG methylation distributions across a number of cell types at varying resolutions, identifying methylation patterns that reflect CpG density, functional chromatin state, and XCI^22–28^. Across the mammalian genome, CpG islands are predominantly unmethylated, with the notable exception of methylation at CpG islands on the Xi^20,29^. However, at genes that escape XCI, which make up approximately 12–20% of human X-linked genes and 3–7% of mouse X-linked genes^30^, CpG islands are often unmethylated on both Xi and Xa^31^. Further, base-resolution analyses of the X chromosome identified a non-CpG methylation (mCH) signature in genes that escape XCI^32,33^.

The characterization of epigenetic profiles on X chromosome presents unique opportunities and key challenges. Firstly, female cells contain both the Xi and Xa within the same nuclear environment. While use of hybrid model systems can distinguish DNA methylation of Xi and Xa in the same cell population^28^, for the vast majority of human (population) and animal studies, Xi and Xa are indistinguishable. Thus, for these studies, observed measurements represent an aggregate of two distinct populations, the Xi and Xa. Second, there is one male X chromosome for every two autosomes. Thus, for sequence-based analyses, the male X chromosome will result in approximately half the read depth of autosomes or the female X chromosome at comparable total sequencing depth. Accordingly, the X chromosome is frequently excluded from female-to-male comparisons of the transcriptome and epigenome^34^.

Here, we employed a methodology to normalize whole genome bisulfite sequencing data for sequencing depth differences across the X chromosome. Using depth-normalized datasets, we characterized sex-specific DNA methylation patterns on the X chromosome at base-resolution in mice of two different genetic backgrounds. We further investigated how these epigenetic modifications relate to dosage compensation of X-linked gene expression. Through these efforts, we confirmed that previous locus-specific findings hold true at a chromosome-wide base-resolution scale and provide new insights about DNA methylation and silencing the Xi.

## Results

### Sex impacts liver gene expression across autosomes and the X chromosome

Sex chromosome dosage is one factor that can influence sex-biased gene expression. From copy number alone, one would expect mammalian X-linked genes to have female-biased expression; however, the mechanisms of dosage compensation have evolved to regulate the expression of the X chromosome^35^. To investigate the extent to which the mechanisms of dosage compensation maintain female-to-male gene expression balance in mouse liver, we analyzed the chromosomal distribution of genes with patterns of sex-biased expression. Using RNA-seq, we profiled the transcriptome of liver tissue from 20-week old male and female mice of the C57BL/6 N (B6) and C3H/HeN (C3) inbred mouse strains (Supplementary Table S1). These two inbred mouse strains exhibit phenotypic differences in liver biology, as they are at opposite ends of the spectrum of spontaneous hepatocellular carcinoma incidence; C3 mice are highly susceptible to develop spontaneous liver tumors as a function of age, while B6 mice are extremely resistant^36^.

**Figure 1:**
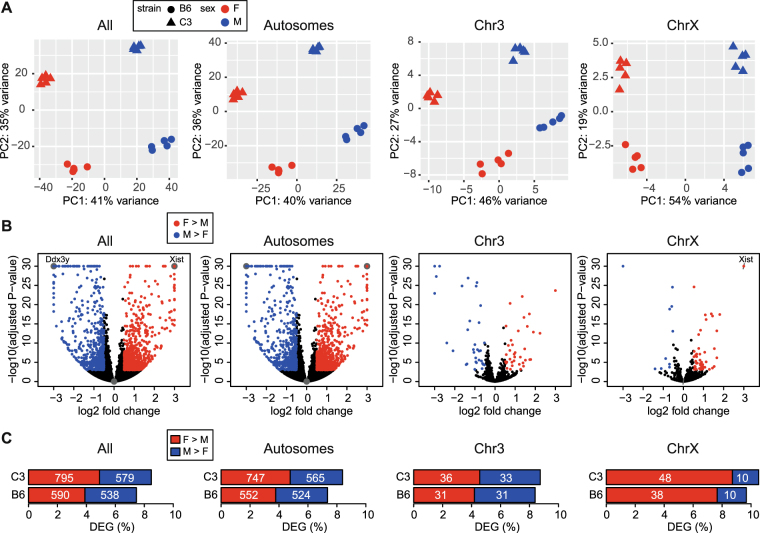
Mechanisms of dosage compensation maintain female to male expression balance for the majority of genes on the X chromosome. (**A**) Principal component analysis (PCA) of gene expression using RNA-seq data from all chromosomes, autosomes, chromosome 3, and chromosome X. Each point represents a liver sample (n = 5 mice per group) with color indicating sex and shape indicating strain. Percent of variance for each principal component (PC1, PC2) is indicated on the axes. (**B**) Volcano plots depicting sex-specific differential expression of genes on all chromosomes, autosomes, chromosome 3, and chromosome X for C3 mice. Significant genes (adjusted P-value < 0.001; |log2 fold change >0.5|) are colored by direction of fold change (Red, female-bias; Blue, male-bias). For visualization purposes, adjusted P-values less than 1.0e-30 were set to 1.0e-30, log2 fold change <−3 were set to −3, and log2 fold change >3 were set to 3. Overlapping points are visualized using sunflower petals. Female-specific *Xist* (chromosome X) and male-specific *Ddx3y* (chromosome Y) are labeled. (**C**) Barplots depicting the percent of female- (red) and male-biased (blue) differentially expressed genes (DEGs) on all chromosomes, autosomes, chromosome 3, and chromosome X. The absolute number of DEGs is indicated on each bar. ChrX, chromosome X; Chr3, chromosome 3.

First, we evaluated to what extent X-linked genes influence overall patterns of similarity between liver transcriptomes of B6 female, B6 male, C3 female and C3 male mice. We performed principal component analysis (PCA) of RNA-seq expression data (5 biological replicates per group) using genes located on all chromosomes, autosomes, the X chromosome, or an autosome of similar size to the X chromosome (chromosome 3) (Fig. 1A). Using expression data from all genes, the first two principal components clearly separated samples by sex and strain. The separation of samples along the first principal component (PC1, 41% variance) predominantly corresponded to sex, while the separation of samples along the second principal component (PC2, 35% variance) predominantly corresponded to strain. Removal of gene expression data from the sex chromosomes (X, Y) from this analysis had minimal impact on overall sample separation. Further, samples similarly separated by sex and strain when only genes on the X chromosome (PC1, 54% variance; PC2, 19% variance) or genes on chromosome 3, presented as a representative autosome, (PC1, 46% variance; PC2, 27% variance) were examined.

Next, to assess expression patterns at the gene level, we performed pairwise comparisons of female versus male gene expression (for the C3 and B6 strains) and C3 versus B6 expression level (for male and female animals). A sortable table of all sex- and strain-specific differentially expressed genes is present in Supplementary Table S2. As expected, known female-specific X-linked genes, including *Xist*, and male-specific Y-linked genes, including *Ddx3y*, were of the most statistically significant genes identified (Fig. 1B). Overall 7.5% and 8.5% of genes exhibited sex-biased expression in the B6 and C3 strains, respectively (Fig. 1C). Approximately half of the identified female-biased genes on the X chromosome and autosomal male- and female-biased genes were common across strains. However, only 20% of male-biased X chromosome genes were common across strains (Supplementary Table S3). Comparable to numbers of sex-biased genes, 9.3% and 10.3% of genes exhibited strain-biased expression in female and male animals, respectively (Supplementary Table S4). A substantial overlap of sex- and strain-biased genes was observed on the X chromosome. Specifically, 20.8% (10/48) of sex-specific X-linked genes in B6, and 15.5% (9/58) of sex-specific X-linked genes in C3 overlapped with strain-biased genes. The fraction of sex-biased genes that overlapped strain-biased genes was even greater on autosomes. Specifically, 39.3% (423/1076) of sex-specific autosomal genes in B6 and 34.3% (450/1312) of sex-specific autosomal genes in C3 overlapped with strain-biased genes.

Within sex-biased genes, the percentage of female-biased genes (3.9% and 4.9% of all genes in the B6 and C3 strains, respectively) was slightly greater than the percentage of male-biased genes (3.6% of all genes in both B6 and C3 strains). Nevertheless, the majority of sex-biased genes were found to be located on autosomes, and the overall proportion of sex-biased genes was not greatly impacted by removal of genes located on sex chromosomes. As expected, autosomal sex-biased genes clustered into particular pathways and biological processes that suggest hormonal regulation. Specifically, the top KEGG pathways identified for sex-biased genes (e.g. steroid hormone biosynthesis, drug metabolism - cytochrome P450, retinol metabolism, chemical carcinogenesis) are highly consistent with the pathways identified in a model of feminization of male mouse liver by persistent growth hormone stimulation^37^ (Supplementary Table S5). However, greater percentages of sex-biased genes were observed on the X chromosome, as compared to autosomes. Specifically, 9.7% and 10.5% of X-linked genes exhibited sex-biased expression in B6 and C3 animals, respectively (Fig. 1C). As expected, the majority of sex-biased genes on the X chromosome were female-biased, exhibiting expression patterns consistent with an associated dosage difference. However, despite the X chromosome dosage difference between males and females, the majority of genes on the X chromosome (89.5% in C3 and 90.3% in B6) did not meet differential significance thresholds, supporting the hypothesis that mechanisms for dosage compensation are robust in mammalian liver.

### Distribution of CpG methylation on the X chromosome reflects XCI

DNA methylation plays a key role in XCI, a process that achieves dosage compensation for X-encoded gene products between male and female mammalian cells. To investigate how chromosome-wide, base-resolution DNA methylation patterns relate to gene expression dosage compensation in mouse liver, we analyzed whole genome bisulfite sequencing (WGBS) datasets from 20-week old female and male mice of the B6 and C3 strains and compared the X chromosome to a representative autosome of similar size (chromosome 3). Libraries from three independent animals per sex for each genotype were utilized to minimize bias due to biological outliers. To control for variations in genetic background and permit cross-strain comparisons, only cytosines with validated sequence context (CG, CHG, CHH) in all genotypes were utilized for downstream analyses (Supplementary Table S6). Finally, to control for read depth differences across sexes, chromosomes and biological replicates, most notably the approximately 2-fold read depth differences between females and males on the X chromosome, a down-sampling approach was employed to normalize the total number of sequencing read counts at validated cytosines on chromosomes X and 3. For each genomic context (CG, CHG, CHH) on chromosomes X and 3, single-cytosine mapped bases at validated sites in each animal were randomly reduced (down-sampled) to reach a total average depth of 5X per validated cytosine, per strand (see Methods). For cytosines in CG context, symmetric strands were combined, resulting in an average depth of 10X per individual animal. Final depth-normalized datasets (3 biological replicates per group) totaled an average depth of 30X per group across CG sites (Supplementary Table S7), meeting the suggested depth requirements to obtain satisfactory sensitivity and specificity in differential methylation discovery for WGBS analyses^38^. The resulting depth-normalized datasets covered at least 89% of validated CpG sites on chromosomes X and 3 at a read depth of at least 10X (Supplementary Table S8).

**Figure 2:**
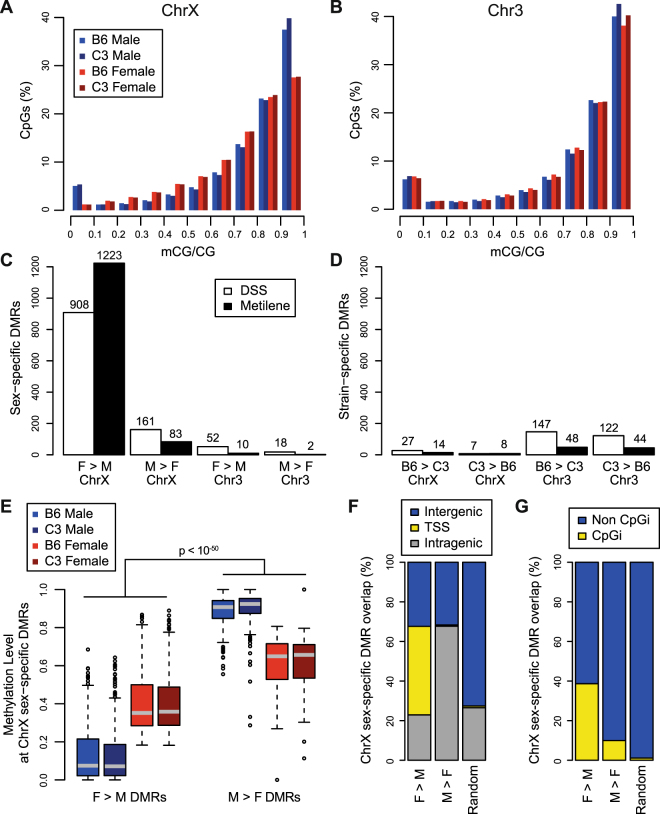
Sex impacts global distribution of CpG methylation on the X chromosome. (**A**) Histogram plot representing frequency distribution of methylation level (mCG/CG) across CpG sites with depth of at least 10x on the X chromosome. Number of CpGs included: B6 Female, 787,051; B6 Male, 793,002; C3 Female, 773,057; C3 Male, 787,158. (**B**) Histogram plot representing frequency distribution of methylation level (mCG/CG) across CpG sites with depth of at least 10x on a representative autosome, chromosome 3. Number of CpGs included: B6 Female, 1,055,125; B6 Male, 1,049,035; C3 Female, 1,014,511; C3 Male, 1,019,177. **(C**) Number of differentially methylated regions (DMRs) on chromosomes X and 3 that distinguish male from female animals using two different tools, DSS and Metilene. DMRs are partitioned by direction, indicating regions with greater methylation level in female mice (F > M) or regions with greater methylation level in male mice (M > F). **(D**) Number of DMRs on chromosomes X and 3 that distinguish B6 from C3 animals. DMRs are partitioned by direction, indicating regions with greater methylation level in B6 mice (B6 > C3) or regions with greater methylation level in C3 mice (C3 > B6). **(E**) Boxplots of CG weighted methylation levels at sex-specific DMRs (using DSS) on the X chromosome for B6 male, C3 male, B6 female, and C3 female mice. Methylation levels at F > M DMRs are significantly less than methylation levels at M > F DMRs, regardless of sex (Mann-Whitney *U*-test). **(F**) Percentage of sex-specific DMRs (using DSS) on the X chromosome that overlap intergenic regions, TSS, or intragenic (non-TSS) regions. **(G**) Percentage of sex-specific DMRs (using DSS) on the X chromosome that overlap CpG island regions or non-CpG island regions. Percentages were calculated based on total DMR count for each type (F > M, M > F, random genomic null region set). ChrX, chromosome X; Chr3, chromosome 3.

Using the depth-normalized datasets, we first assessed chromosome-wide CpG methylation level distributions for male and female animals of each strain using X chromosome and chromosome 3. Consistent with previous genome-wide base-resolution assessments of CpG methylation, the majority of CpG sites assayed were identified as highly methylated (mCG/CG >0.6), regardless of chromosome location, sex, or genetic strain. However, distinct sex-specific distributions were observed on the X chromosome (Fig. 2A), but not on chromosome 3 (Fig. 2B). As expected, the bimodal distribution of CpG methylation observed on the male X chromosome is similar to that of an autosome. However, compared to male CpG methylation distributions on the X chromosome, female animals exhibited decreased frequency of fully unmethylated CpG sites (mCG/CG, 0–0.1) and decreased frequency of fully methylated sites (mCG/CG, 0.9–1.0). Concomitant with the differences at the extremes of the distribution on the X chromosome, females exhibited an increased frequency of CpG sites with intermediate levels of methylation (mCG/CG, 0.2–0.8), compared to males.

Next, we sought to characterize the specific genomic regions that contribute to the observed sex-specific CpG methylation distributions on the X chromosome. We utilized two established tools, DSS^39^ and Metilene^40^, to detect sex- and strain-specific differentially methylated regions (DMRs) on chromosomes X and 3 (Supplementary Table S9). We compared DMR calls using the new framework presented here (using depth-normalized datasets) and the normal pipeline (using full datasets), and while the majority of DMR calls on the X chromosome are similar using both DSS and Metilene (82.8–95.1% overlap), using the new framework may reduce false positive calls resulting from sequencing depth biases (Table 1). Consistent with our observed chromosome-wide methylation distributions, the sex-specific DMRs identified on the X chromosome far outnumbered the sex-specific DMRs identified on chromosome 3 (Fig. 2C). The vast majority of sex-specific DMRs on the X chromosome exhibited greater methylation levels in females (N = 908, 84.9%, DSS; N = 1223, 93.6%, Metilene). However, DMRs exhibiting greater methylation levels in males on the X chromosome (N = 161, DSS; N = 83, Metilene) far outnumbered DMRs exhibiting greater methylation levels in males on chromosome 3 (N = 18, DSS; N = 2, Metilene). Further, the number of sex-specific DMRs identified from both strains on the X chromosome (N = 1069, DSS; N = 1306, Metilene) was far greater than the number of strain-specific DMRs identified from both sexes on either chromosome 3 (N = 269, DSS; N = 92, Metilene) or chromosome X (N = 34, DSS; N = 22, Metilene; Fig. 2D). Female to male methylation differences at sex-specific DMRs on the X chromosome were largely consistent between strains (Supplementary Fig. S1). However, strain-specific DMRs were more frequently observed on chromosome 3 as compared to the X chromosome, which cannot be explained by the difference in chromosome length (159.6 Mb, chromosome 3; 166.7 Mb, chromosome X).

**Table 1:** Comparison of sex-specific DMR calling on X chromosome using full and normalized datasets.

	X chromosome DMRs using all data			X chromosome DMRs using normalized data		
Tool	#	overlap ‘normalized’ DMRs		#	overlap ‘all’ DMRs	
DSS	1124	931	82.8%	1069	945	88.4%
Metilene	1342	1134	84.5%	1306	1242	95.1%

Female versus male DMR calls for X chromosome were generated using both the new framework presented here (using normalized datasets) and the normal pipeline (using full datasets) with both the DSS and Metilene tools. Numbers of DMRs identified are listed along with numbers and percentages of overlapping DMRs.

Depending on the direction of methylation difference, sex-specific DMRs on the X chromosome exhibited different methylation levels. Loci on the X chromosome with greater methylation levels in females (F > M DMRs) predominantly occurred at regions that are unmethylated in males. Loci on the X chromosome with greater methylation levels in males (M > F DMRs) predominantly occurred at regions that are highly methylated in males. Regardless of the sex, methylation levels at F > M DMRs were significantly lower than methylation levels at M > F DMRs on chromosome X (Fig. 2E; p < 10^−50^, Mann-Whitney *U*-test). This was not observed at sex-specific DMRs on chromosome 3 or at strain-specific DMRs on chromosomes X or 3 (Supplementary Fig. S2). These data concurred with the observed differential sex-specific chromosome-wide distributions and support that loci with extreme methylation values in either direction (mCG/CG approaching 0 or 1.0) in males are represented at intermediate methylation levels (mCG/CG 0.2 – 0.8) in females.

Further, depending on the direction of methylation difference, sex-specific DMRs on the X chromosome exhibited differential location distributions relative to genomic features. F > M DMRs were enriched at TSSs on the X chromosome, as 44.7% of F > M DMRs overlapped a TSS, compared to 0.6% of M > F DMRs or 0.9% of length-matched null control regions. On the other hand, M > F DMRs were enriched in gene bodies on the X chromosome, as 67.7% of M > F DMRs overlapped non-TSS intragenic regions, compared to 22.9% of F > M DMRs or 26.5% of random control regions (Fig. 2F). Likewise, F > M DMRs were enriched at CpG islands on the X chromosome, as 38.7% of F > M DMRs overlapped a CpG island, compared to 9.9% of M > F DMRs or 1.1% of length-matched null control regions (Fig. 2G). Approximately one-third (32.7%) of F > M DMRs on the X chromosome overlapped both a CpG island and TSS. Enrichment at TSSs or CpG islands was not observed at sex-specific DMRs on chromosome 3 or at strain-specific DMRs on chromosome 3 or chromosome X (Supplementary Fig. S3). Together, the observed base-resolution distribution of CpG methylation on the X chromosome concurs with longstanding observations of XCI at promoter-proximal CpG islands^20^.

However, while sex-specific methylation differences at CpG islands and TSSs on the X chromosome are clear, F > M DMRs on the X chromosome are not limited to these regions. 22.9% and 32.4% of F > M DMRs occur at intragenic and intergenic regions, respectively. We expect many non-TSS F > M DMRs to occur at enhancer regions, and indeed most (59.4% of non-TSS F > M DMRs) do overlap with enhancer-associated H3K4me1 peaks^41^ in the male liver (Supplementary Fig. S4).

### Sexual dimorphisms of DNA methylation are prominent at promoter-proximal CpG islands

**Figure 3:**
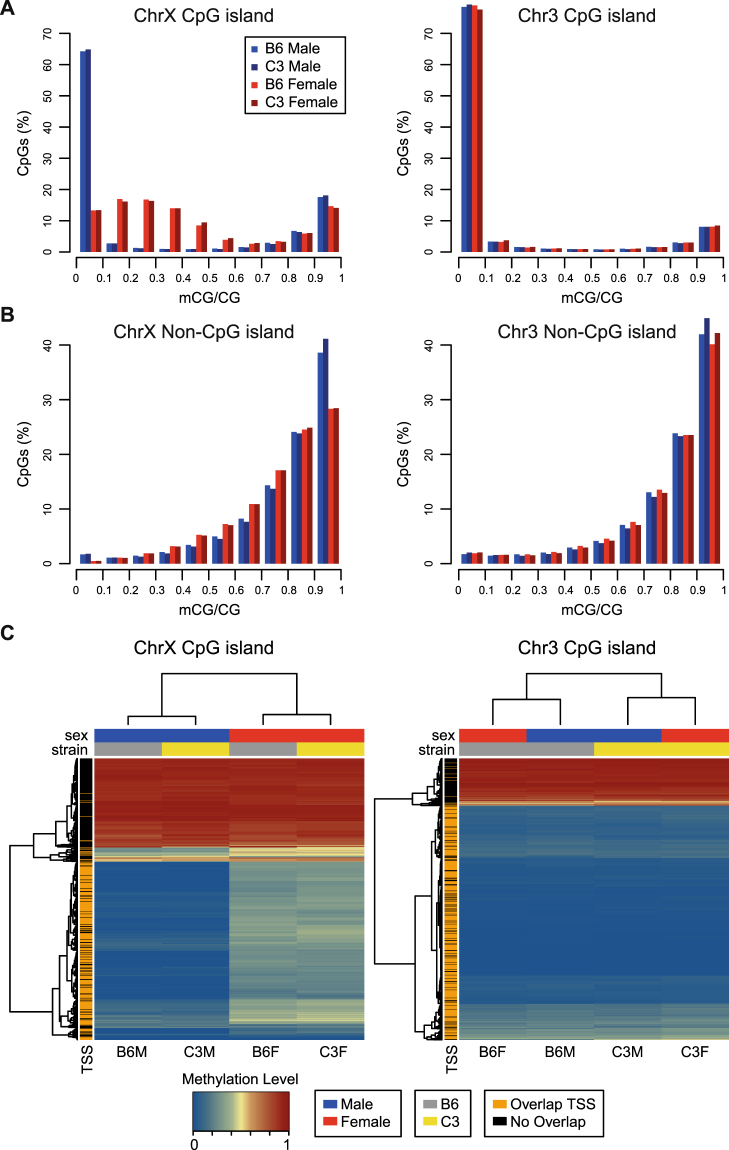
Sexual dimorphisms at low methylation levels (0–50%) on the X chromosome largely reflect differences at promoter-proximal CpG islands. (**A**) Histogram plots representing frequency distributions of methylation level (mCG/CG) across only CpG sites located in CpG islands on chromosomes X and 3, with read depth of at least 10x. Number of CpGs included on the X chromosome: B6 Female, 44,430; B6 Male, 42,534; C3 Female, 40,345; C3 Male, 44,405. Number of CpGs included on chromosome 3: B6 Female, 67,135; B6 Male, 61,067; C3 Female, 59,010; C3 Male, 63,935. (**B**) Histogram plots representing frequency distributions of methylation level (mCG/CG) across only CpG sites located outside CpG islands on chromosomes X and 3, with read depth of at least 10x. Number of CpGs included on the X chromosome: B6 Female, 742,621; B6 Male, 750,468; C3 Female, 732,712; C3 Male, 742,753. Number of CpGs included on chromosome 3: B6 Female, 987,990; B6 Male, 987,968; C3 Female, 955,501; C3 Male, 955,242. (**C**) Heatmaps of methylation levels for CpG island regions on chromosomes X and 3. Rows represent CpG islands containing at least a minimum number of validated CpG sites (calculated as (length/16)*0.65) and with an average minimum read depth of at least 10x across all samples (n = 596 for chromosome X and n = 740 for chromosome 3). The methylation scale indicates the weighted methylation level of each island: blue, unmethylated; yellow, intermediate; red, methylated. Columns represent animal groups. Row and column dendrograms depict unsupervised clustering based on row or column means, using Euclidian distance with complete linkage clustering. Row sidebar indicates whether or not each CpG island overlaps a TSS. Column sidebars indicate sex and strain. ChrX, chromosome X; Chr3, chromosome 3.

To further investigate how the landscape of DNA methylation on the mammalian X chromosome reflects CpG density, we examined methylation patterns at CpG islands in our model. Using a subset of the depth-normalized datasets, we assessed CpG methylation level distributions for only CpG sites falling within CpG island boundaries on chromosomes X and 3 in male and female animals of each strain (Fig. 3A). Consistent with our differential methylation analyses, sex-specific distributions were observed on the X chromosome, but not on chromosome 3. For CpG islands on the control autosome and male X chromosome, the majority of CpG sites assayed were identified as unmethylated (mCG/CG < 0.1). In contrast, the prominent population of unmethylated CpG sites was not observed on the female X chromosome. Instead, a broader distribution of CpG sites with methylation level (mCG/CG) ranging from 0 to 0.6 was observed. Rather than appearing as a focused peak at mCG/CG 0.5, which would result from a population in which half of the sites are unmethylated and the other half fully methylated, the peak of this distribution occurred at methylation levels (mCG/CG) ranging from 0.1 to 0.3. When examining the remainder of CpG sites, i.e., those sites not falling within a CpG island region, the resulting distribution was largely unimodal (Fig. 3B). However, compared to male CpG methylation distributions on the X chromosome, females exhibited an increased frequency of CpG sites with intermediate levels of methylation (mCG/CG, 0.2–0.8), as compared to males.

To understand how the single site methylation distribution relates to regional CpG island methylation levels, we compared weighted methylation levels for CpG islands on chromosomes X and 3 for male and female mice of both strains (Fig. 3C). First, on both chromosomes X and 3, most CpG islands can be classified into two primary groups by regional methylation level: CpG islands that are predominantly methylated in all groups, and CpG islands that are predominantly unmethylated. Whether or not the CpG island overlaps a TSS is largely predicted by its assignment to one of these two groups (Fig. 3C; see row dendrogram and color bar). Second, CpG island methylation level segregated animals by sex on the X chromosome, but by strain on chromosome 3 (Fig. 3C; see column dendrogram and color bar). Third, regional methylation levels for CpG islands that are unmethylated on the male X chromosome did not approach 50% in female animals. Consistent with single site methylation level data, these results suggest that CpG islands on the Xi are not fully methylated.

### Sexual dimorphisms of DNA methylation extend beyond CpG island boundaries

**Figure 4:**
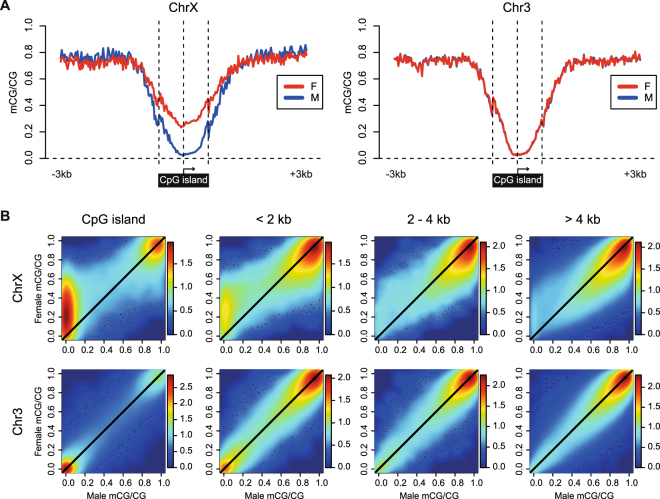
Sexually dimorphic methylation differences on the X chromosome are observed beyond CpG island boundaries. (**A**) Metaplots of CpG methylation level at CpG islands overlapping exactly one TSS on chromosome X (n = 275) and chromosome 3 (n = 429) for C3 animals (Red, female; Blue, male). CpG islands are oriented to the direction of transcription of the overlapped TSS, and the upstream and downstream CpG island edges are independently scaled relative to the TSS. An additional 3 kb to either side of the CpG island (unscaled) is included. (**B**) Smoothed kernel density scatterplots comparing male and female single-site methylation levels on chromosomes X and 3, by proximity to CpG islands. C3 female mCG/CG (y-axis) versus C3 male mCG/CG (x-axis) is plotted for CpG sites with depth of at least 10x in each sample located in CpG islands, within 0–2 kb from a CpG island edge, within 2–4 kb from a CpG island edge, or greater than 4 kb from the nearest CpG island. Points to the upper-left of the diagonal reflect CpGs with greater female methylation and points to the lower-right of the diagonal reflect CpGs with greater male methylation. Number of CpGs included on the X chromosome: CpG island, 39,050; <2 kb, 24,571; 2–4 kb, 18,336; >4 kb, 680,325. Number of CpGs included on chromosome 3: CpG island, 56,661; <2 kb, 31,576; 2–4 kb, 27,092; >4 kb, 883,370. Density color keys are shown to the right of each plot. ChrX, chromosome X; Chr3, chromosome 3.

We next examined how sexually dimorphic DNA methylation patterns relate to CpG island boundaries and TSSs. To do this, we performed a meta-analysis of single site methylation at and around CpG islands located on chromosomes X or 3 that overlap exactly one TSS. Consistent with results presented in Fig. 3, sexually dimorphic methylation was clearly apparent within CpG island boundaries on the X chromosome, while metaplots for male and female animals were nearly identical on chromosome 3 (Fig. 4A, Supplementary Fig. S5). Methylation differences were observed to a lesser extent at TSSs not located in CpG islands (Supplementary Fig. S6). Similar to CpG island TSSs on chromosome 3, methylation approaches zero at CpG island TSSs on the male X chromosome and increases towards each CpG island edge. Interestingly, sex-specific methylation differences are not limited to CpG island boundaries, and extend outward into CpG island shores, resulting in methylation differences in the intermediate range (mCG/CG 0.3–0.7).

To extend this analysis, we compared all female and male single site CpG methylation levels on chromosomes X and 3 by proximity to CpG island boundaries (Fig. 4B, Supplementary Fig. S5). Indeed, CpG sites with greater methylation levels in females (upper left of diagonal) were observed at CpG island shores (<2 kb from CpG island edge) and were often present at intermediate methylation levels (mCG/CG 0.3–0.7). On the other hand, skewing towards CpG sites with greater (mCG/CG 0.7–1) methylation level in males (lower right of diagonal), although less pronounced, was observed at CpG island shores, CpG island shelves (2–4 kb from CpG island edge), and open sea regions (>4 kb from CpG island edge). Together, these results are consistent with the observed enrichment of F > M DMRs at promoter-proximal CpG islands and with the enrichment of M > F DMRs at gene body regions.

Next, we examined the relationship between methylation levels at gene body regions and transcription levels. For genes expressed in both sexes, intragenic methylation levels (mCG/CG) are significantly higher in males than in females (p = 3 × 10^−6^, Mann-Whitney U-test, Supplementary Fig. S7). Further, in male animals, intragenic methylation levels for expressed genes are higher than levels for silent genes (p = 3 × 10^−3^, Mann-Whitney U-test, Supplementary Fig. S7). Genes that are silent in both sexes (i.e. silent on both the Xa and Xi) fail to show a significant difference in intragenic methylation levels. Together, these data provide supporting evidence for a relationship between transcription levels and gene body methylation levels.

### Exceptions to the rule: escape from XCI

While DNA methylation appears to be a part of the mechanism for dosage compensation for the majority of genes on the X chromosome, exceptions to XCI were observed. We identified a group of X-linked female-biased genes that exhibit expression patterns consistent with an associated dosage difference. Lister *et al*. previously reported a non-CpG methylation (mCH) signature that identifies genes that escape XCI in neurons, a cell type in which non-CpG methylation accumulates at high levels^32^. Schultz *et al*. evaluated the prevalence of mCH in multiple additional human tissues and found the fraction of methylated cytosines in CH context to ranges from 0–0.4%, as compared to over 8% for neurons^33^. Further, they demonstrated that the mCH signature identifies escape genes in several human tissues, including adrenal, aorta, esophagus, fat, gastric, pancreas, small bowel, and spleen^33^.

**Figure 5:**
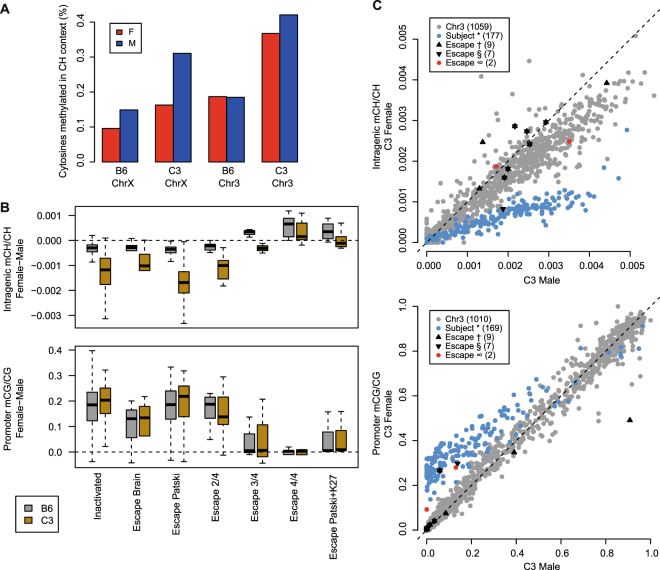
Non-CpG methylation is enriched in genes escaping XCI in mouse liver. (**A**) The total fraction of methylated cytosines in CH (non-CpG) context by sex and strain for chromosomes X and 3 (Red, female; Blue, male). (**B**) Sex differences in intragenic mCH/CH and promoter mCG/CG distinguish escaper genes from inactivated genes. Boxplots of sex differences (female – male) in intragenic mCH/CH and promoter mCG/CG at genes with previously demonstrated XCI status in multiple mouse cell types. Classifications include genes subject to XCI in both brain and Patski cells (n = 177), brain-specific escapers (n = 6), Patski-specific escapers (n = 52), genes escaping in 2 of 4 (n = 9), 3 of 4 (n = 3), or 4 of 4 (n = 6) cell types analyzed by Berletch *et al*.^42^, or genes escaping in the Patski cell line and depleted of H3K27me3 in adult mouse liver (n = 7), as demonstrated by Yang *et al*.^43^. (**C**) Gene-level scatterplots comparing C3 male and C3 female intragenic mCH/CH and promoter mCG/CG for mouse genes by XCI status. Mouse genes reported as inactivated or escapers: Berletch *et al*. subject to XCI in both brain and Patski cells (*), Berletch *et al.* escape in at least 3 of 4 cell types (†), Yang *et al*. escape in Patski cells and depleted of H3K27me3 in adult liver (§). Predicted escaper genes (∞) and autosomal genes (Chr3) are also indicated.

Here, we assessed to what extent the mCH signature held true for genes likely to escape XCI in mouse liver tissue from two different genetic backgrounds. In mouse liver, the total fraction of methylated cytosines in a CH context (0.1–0.4%) was highly consistent with the range observed in non-neuronal human tissues, albeit much lower than mCH fractions in neurons (Fig. 5A). In our study, the fraction of mCH varied by sex and strain for chromosomes X and 3; the fraction of methylated CH sites was approximately 2-fold greater in the C3 strain than in the B6 strain for the comparable sex and chromosome. Furthermore, consistent with previous reports^32^, male animals exhibited a higher fraction of methylated CH sites on the X chromosome than female animals of the same strain, presumably due to effects of XCI.

To classify inactivation status of X-linked genes, we relied on two published reports that surveyed allele-specific gene expression of X-linked genes in mouse tissues and cells with skewed XCI^42,43^. Genes reported to escape XCI were categorized based on either the level of variability between four mouse cell types^42^ or the overlap with H3K27me3-depleted regions in mouse liver^43^ (see Methods for detailed description). Despite strain-specific differences in overall fractions of cytosines methylated in CH context, sex differences in intragenic mCH/CH and promoter mCG/CG distinguished escaper genes from inactivated genes in both strains (Fig. 5B). However, sex differences were not apparent when we analyzed intragenic mCG and promoter mCH (Supplementary Fig. S8). At the individual gene level, greater intragenic mCH levels and reduced promoter mCG levels distinguished genes escaping XCI from genes subject to XCI when comparing females to males (Fig. 5C, Supplementary Fig. S9). Despite a lower overall fraction of mCH sites on the X chromosome in the B6 strain, intragenic mCH levels were predictive of XCI status in both strains (Supplementary Fig. S10). Using promoter mCG and intragenic mCH levels of genes on the X chromosome (Supplementary Table S10), we classified escapers of XCI in each strain (Table 2, Supplementary Table S11). The genes identified in the current study as liver escapers are frequently reported to escape XCI in other mouse cell types, including trophoblast stem cells (TSCs)^44^, neural progenitor cells (NPCs)^15,45^, Patski cells^42^, spleen^42^, ovary^42^, and brain^28,42,46^ (Table 2). Based on our data, we predict that non-CpG methylation is enriched in genes that escape XCI in mouse liver.

**Table 2:** Summary of genes predicted to escape from XCI in mouse liver based on DNA methylation data.

Gene	Analysis Set	Liver C3	Liver B6	Patski^42^	Spleen^42^	Ovary^42^	TSCs^44^	NPCs^15^	NPCs^45^	Brain^42^	Brain^46^	Brain^28^
*Ddx3x*	†,§	Escape	Escape	*	*	*		*	*	*	*	*
*Eif2s3x*	†,§	Escape	Escape	*	*	*	*	*		*	*	*
*Firre*	†,§	Escape	Escape	*	*	*		*	*			*
*Kdm5c*	†,§	Escape	Escape	*	*	*	*	*	*	*	*	*
*Kdm6a*	†,§	Escape	Escape	*	*	*	*	*	*	*	*	*
*Pbdc1*	†,§	Escape	Escape	*	*	*	*	*	*			*
*5530601H04Rik*	†	Escape	Escape	*	*	*	*		*	*	*	*
*Ftx*	†	Escape	Escape		*	*	*		*	*		*
*Xist*	†	Escape	Escape	*	*	*	*	*	*	*	*	*
*Mid1*	§	Subject	Subject	*			*	*	*		*	
*4933407K13Rik*	∞	Escape	Subject									‡
*G530011O06Rik*	∞	Subject	Escape				*					
*Utp14a*	∞	Escape	Subject		*	*	*		*			

Mouse genes reported as escaping from or subject to XCI in the C3 and B6 mouse strains, according to the data in this study. Analysis set designates genes reported by Berletch *et al*.^42^ escaping in at least 3 of 4 cell types (†), genes reported by Yang *et al*.^43^ escaping in Patski cells and depleted of H3K27me3 in adult liver (§), or genes not included in the training set (∞). Genes identified to escape XCI in other studies are indicated with an asterisk (*). Mouse cell types in the comparison studies include Patski cells, spleen, ovary, trophoblast stem cells (TSCs), neural progenitor cells (NPCs), and brain cells. Also noted is a gene previously identified to be CH-hypermethylated, located at the *Dxz4* macrosatellite (‡).

## Discussion

Here, we utilized a high-depth whole genome bisulfite sequencing dataset to draw insights on a longstanding topic of interest, the role of DNA methylation in XCI, in a mouse model system widely used for toxicological assessments. The depth and resolution of our dataset empowered an investigation of the generalizability of decades of gene-by-gene findings, while addressing unique depth considerations for sequence-based analysis of female-to-male comparisons on the X chromosome. Based on the analyses we have developed and report here, the general models of DNA methylation on the Xi previously described are solid and appear to appropriately predict XCI status. However, while confirming many longstanding observations, several additional insights were made regarding X-chromosome-wide methylation distributions, maintenance of DNA methylation at CpG islands, and methylation in gene body regions.

First, using our high-resolution WGBS datasets, we provide perspective on chromosome-wide methylation distributions that may inform animal or human population studies comparing epigenomes of male and female subjects. Consistent with a previous finding that global levels of methylation on the two female X chromosomes did not significantly differ^47^, the majority of CpG sites were consistently highly methylated on the X chromosome regardless of presence of the Xi (Fig. 2A). This base-resolution chromosome-wide distribution starkly differs from distributions of sex-specific DNA methylation patterns on the human X chromosome that have been assessed using Illumina Infinium HumanMethylation450 (450 K array) BeadChips, which cover approximately 1% of CpG sites on the human X chromosome and are biased towards TSSs^26,27^. CpG methylation frequency distributions of X-linked 450 K array probes in female and male humans look quite different than global distributions presented here. Specifically, frequency distribution analyses of 450 K array probes located on the X chromosome present a bimodal distribution for males (2 distinct peaks at β ≈ 0 and β ≈ 1) and a multimodal distribution in females (3 peaks at β ≈ 0, β ≈ 0.5, and β ≈ 1)^26,27^. The female X-linked 450 K array probe methylation distributions more similarly resemble methylation distributions resulting from analysis of CpG island CG sites (Fig. 3A) than methylation distributions resulting from analysis of all CG sites on X chromosome (Fig. 2A).

In the mammalian genome, CpG islands are predominantly unmethylated regions of DNA, with the only major exception being methylation of CpG islands of the Xi. Consistent with previous studies, a large proportion of female versus male mCG differences on the X chromosome occur at CpG islands. However, our analysis provides additional insights relating to maintenance of DNA methylation at CpG islands. First, sex-specific DNA methylation differences on chromosome X are not limited to CpG island boundaries. While sex differences at regions with low methylation levels (mCG/CG 0–0.3) on the X chromosome reflect differences at promoter-proximal CpG islands subject to XCI, sexually dimorphic differences at intermediate methylation levels (mCG/CG 0.3–0.7) were often observed at regions adjacent to CpG islands. Second, the results presented here suggest that CpG islands on the Xi are not fully methylated. This is consistent and widespread across CpG islands on the Xi (Fig. 3C). Interestingly, the peak of the female methylation distribution of CpG sites within an island occurs from methylation level (mCG/CG) ranging from 0.1 to 0.3 (Fig. 3A). This would not be the expected distribution if Xa were unmethylated and Xi were fully methylated, in which a sharp peak around 0.5 would be expected. The observations here may reflect a general rule for silencing a CpG island, in that a biochemical threshold exists for blocking access to factors that keep DNA unmethylated. Visual inspection of individual CpG islands in our data suggests that locations near TSSs exhibit methylation levels close to 50%. We speculate that the threshold for maintenance of the silent state could be location-based, in that methylation of specific cytosines are required for silencing. Rigorous testing of this model will require use of genetic models in which X inactivation is non-random and allelic expression and methylation can be unambiguously determined.

Next, the results presented here inform on the relationship between transcription and DNA methylation patterns in gene body regions. In keeping with the predictions made by Jones over a decade ago^48^, the results here support a paradox in which DNA methylation in the promoter is inversely correlated with gene expression, whereas methylation in the gene body is positively correlated with gene expression^49^. This pattern was exemplified at DMRs on the X chromosome, comparing males with one Xa to females with one Xa and one Xi. In general, greater methylation was observed in gene bodes of males (all actively transcribing) than females (only half actively transcribing). Further, genes that were not expressed in either sex (i.e. silent on both the Xa and Xi) failed to show a significant difference in intragenic methylation levels. Conversely, greater methylation was observed in promoters of females as compared to males.

In addition to assessing methylation at cytosines in the CpG context, the depth and resolution of our dataset allowed us to query the generalizability of recently described non-CpG gene body methylation signatures^32,33^ on the X chromosome in the mouse liver. Compared to brain, liver has far less non-CpG methylation. However, though less than 0.4% of all non-CpG cytosines on the X chromosome were methylated above background levels, intragenic mCH was enriched for a subset of genes that were previously demonstrated to escape XCI in mouse cells. Indeed, we found that intragenic mCH/CH could be used to predict genes that escape XCI in mouse liver. In addition to classifying nine common escape genes between B6 and C3 liver, we sought to identify strain-specific escapers, as such genes have been previously suggested^44^. According to the methodology used in this study, we identified three strain-specific escape genes; 4933407K13Rik and Utp14a escape specifically in C3 and G530011O06Rik escapes specifically in B6. All three of these genes have been previously implicated as escapers in other mouse cell types^28,50,51^.

Finally, the analyses presented here provide insight on methodological aspects of assessing DNA methylation across the X chromosome. Currently, coverage recommendations for methylation analysis by whole-genome bisulfite sequencing are based on autosomes^38^. However, when sequencing male (XY) and female (XX) samples to an equivalent total genomic coverage, the resulting depth of reads mapping to the male X chromosome is approximately half that of autosomes or the female X chromosome. Here, we provide a general framework for performing sample comparisons that have different chromosome numbers. To ensure comparisons are valid across sexes and genomic background, as well as to minimize impact of individual-to-individual variation, cytosine reads at sites with validated genomic context are randomly down-sampled to levels of the lowest coverage animal. Functionally, this process doubles the minimum genomic coverage recommendations if female-to-male or male-to-male comparisons of the X chromosome are to be included in a bisulfite sequencing analysis. In addition to sex chromosome analysis, this process may be useful for other situations where chromosome ploidy may influence the outcome of genomic analysis, for instance in comparisons of cancer versus normal cells.

Together, our analyses provide insight on the underlying DNA methylation landscape in mouse liver and emphasize the importance of understanding the variability of epigenetic and gene expression patterns between sexes. However, moving forward with more widespread evaluation of sex differences^52^, it is important to consider underlying methylation differences between males and females on the X chromosome.

## Methods

### Animal care

Female and male C57BL/6 N and C3H/HeN mice were obtained from Taconic Farms, Inc. (C57BL/6NTac, C3H/HeNTac). Animals were housed in an AALAC accredited facility at Alion Science and Technology (Durham NC, Study # 2015–104–134). Animals were single housed in polycarbonate cages containing absorbent heat-treated hardwood bedding (NEPCO, Warenburg, PA), with male and female mice maintained in separate rooms. Cotton fiber nestlets (Ancare Corp., Bellmore, NY) were supplied to mice for environmental enrichment. Animals were maintained in climate controlled rooms (18–26 °C; 35–65% humidity) on a 12:12 hr light:dark cycle. Animals were housed in a pathogen-free environment, and no pathogens were identified in sentinel testing of serum and feces. To simulate dietary conditions of National Toxicology Program (NTP) breeding, NIH-31 diet (Harlan Teklad, Indianapolis, IN) was provided until 17 weeks of life and then switched to NTP2000 (Zeigler Bros., Gardners, PA) beginning at 18 weeks of age. Water (City of Durham, NC) and food were provided *ad libitum*. At 20 weeks, all animals were euthanized by carbon dioxide asphyxiation, and death was confirmed by exsanguinations via the caudal vena cava. All animal studies were approved by the Alion Animal Care and Use Committee. All procedures were in compliance with the Animal Welfare Act Regulations, 9 CFR 1–4 with handling and treatment according to the *Guide for the Care and Use of Laboratory Animals*^53^.

### RNA extraction

Total RNA was extracted from fresh liver tissues, with 5 biological replicates per group. Time of tissue collection was standardized over a 4-hour period, with necropsies starting at 8 AM local time. The left lateral lobe was rapidly removed, rinsed with phosphate buffered saline, immediately homogenized in Trizol (Thermo Fisher Scientific) using a Kimble motorized pellet pestle, and phase separation was performed according to the manufacturer’s instructions. The Trizol aqueous phase was then purified using Acid Phenol:Chloroform (Ambion) according to the manufacturer’s instructions. An equal volume of 50% ethanol was added to the aqueous layer and the RNA was purified using the RNeasy Mini Kit (Qiagen) with RNase-Free DNase treatment (Qiagen), according to the manufacturer’s instructions. RNA integrity was assessed using the Agilent Bioanalyzer with the RNA 600 Pico Kit (Agilent). The RNA Integrity Number (RIN) for total RNA samples ranged from 9.3 to 9.7.

### RNA-seq library construction and sequencing

Indexed paired-end libraries were constructed for each total RNA sample using the TruSeq Stranded Total RNA Library Prep Kit with Ribo-Zero Gold (Illumina), according to the manufacturer’s instructions. Libraries were randomly distributed to three pools, and each pool was sequenced using the Illumina HiSeq 2500 Sequencing System, run as indexed 125 base paired-end reads, at the NIH Intramural Sequencing Center (NISC). At least 42 million raw read pairs per library were generated.

### RNA-seq data processing and alignment

Initial quality control checks on raw sequence data were performed using FastQC v0.11.4. Datasets were filtered to include read pairs with average base quality score of at least 20. 3′ Illumina universal adapter sequences were removed from each mate using Cutadapt v1.9.1^54^ with the parameters “-a AGATCGGAAGAG -O 3”. Following adapter trimming, appropriate pairing of reads was confirmed and read pairs less than 30 nt were excluded. Trimmed and filtered RNA-seq data were aligned to the mouse genome (mm9/NCBI build 37) using STAR v2.5.1b^55^ with the following parameters: “--runMode alignReads --outSAMtype BAM Unsorted SortedByCoordinate --outMultimapperOrder Random --outSAMattrIHstart 0 --outFilterType BySJout --alignSJoverhangMin 8”. A summary of read pair filtering and mapping is included in Supplementary Table S1. Genomic features were obtained from RefSeq annotations (February 8, 2016). Reads were summarized based on the RefSeq annotations using the featureCounts read quantification program of the Subread v1.5.0-p1 package^56^ with default parameters except “-s2 -p” such that reads were counted at the meta-feature level, and multimapping or multi-overlapping reads were not counted. A matrix of raw counts per gene for each sample (n = 20) was compiled.

### Differential expression analyses

Differential expression analyses were carried out using DESeq2 v1.10.1^57^. A DESeq2DataSet (dds) object was constructed from the matrix of counts using ‘DESeqDataSetFromMatrix’ utilizing a multi-factor design to test the ‘sample’ condition and control for ‘pool’ (design = ~pool + sample), and standard differential expression analysis was performed, ‘DESeq(dds)’. Pairwise differential comparisons of female versus male for each strain and C3 versus B6 for each sex (Supplementary Table S2) were extracted by calling the ‘contrast’ argument of the ‘results’ function with default parameters.

### Statistical and graphical analyses of gene expression data

Statistical and graphical analyses of gene expression were conducted using R version 3.4.1^58^. To generate PCA plots, normalized count data from DESeq2 were transformed by applying a variance stabilizing transformation (VST) using the ‘varianceStabilizingTransformation’ function with default parameters. Subsets of the VST-transformed matrix, reflecting all genes (N = 25,444) or genes on autosomes (N = 23,974), chromosome 3 (N = 1,165), and chromosome X (N = 1,281), were generated using chromosome location annotations. For each subset, row variance was calculated using the ‘rowVars’ function of the ‘genefilter’ package, and the top 10% of genes, selected from each respective group by highest variance across all samples, were utilized for PCA analysis. PCA analysis was performed using the ‘prcomp’ function of the ‘stats’ package^58^. The percent of the total variance that is explained by the principal components in each direction was calculated and is listed on the axes. PCA plots were generated using ‘ggplot’ of the ‘ggplot2’ package^59^. For volcano plots, DESeq2 pairwise differential comparisons of female versus male were filtered to include only genes passing default DESeq2 independent filtering criteria and excluding genes annotated as ‘chrN_random’. The total numbers of genes represented in each plot are as follows: all, 16,199; autosomes, 15,644; chromosome 3, 789; chromosome X, 551. For visualization purposes, adjusted P-values less than 1.0e-30 were set to 1.0e-30, log2 fold change less than −3 were set to −3, and log2 fold change greater than 3 were set to 3. Using the ‘sunflowerplot’ function of the ‘graphics’ package, −log10(adjusted P-value) was plotted against log2 fold change for each gene, such that overlapping points are visualized using sunflower petals. Thresholds for significance were defined as adjusted P-value < 0.001 and |log2 fold change >0.5|.

### WGBS data processing and normalization

WGBS datasets from females and males of the B6 and C3 strains were processed and aligned to the mm9 reference genome (NCBI 37) as previously described (Grimm *et al*., submitted). The observed bisulfite conversion rate was at least 99.4% (mean, 99.8%), as assessed by read pairs mapped to Enterobacteria phage λ (Supplementary Table S12). For downstream analyses, reads mapping to chromosomes X and 3 were utilized. Chromosome 3 was selected as the autosomal control for chromosome X due to similarity in size and CpG content. A genomic context validation strategy was implemented to identify mm9 CpG sites that are within the same genomic context (CG, CHH, CHG) in all genotypes (mm9, B6, C3; Supplementary Table S6), as previously described (Grimm *et al*., submitted). All downstream analyses were restricted to cytosines with validated genomic context. Additionally, data were normalized to account for read depth differences across sexes, chromosomes and biological replicates. Aligned cytosine reads mapping to validated sites were randomly downsampled to an average depth of 5X per strand for each animal (3 animals per genotype). For cytosines in CG context, symmetric strands were combined, resulting in an average depth of 10X per individual animal. Final working datasets totaled 30X average depth (combined strands) per genotype for cytosines in CG context and 15X average depth (per strand) per genotype for cytosine in CH context (Supplementary Table S7). Biological replicate information was utilized for differential methylated region detection, and replicates were merged for all other downstream analyses. For analyses of single-site CG methylation levels, an additional read depth filter was applied such that only CpGs with depth of at least 10X (combined strands) were included. The total number of CG sites with depth ≥10X are listed in Supplementary Table S8 and represent >80% of mm9 CpGs and >89% of validated CpGs (mm9, B6, C3) on chromosomes X and 3.

### Comparative DNA methylation analyses

Differentially methylated regions were identified using two methodologies, DSS^39^ and Metiline^40^. Using depth-normalized CpG data, pairwise comparisons of female versus male for each strain and C3 versus B6 for each sex were conducted using each methodology. For regional CG or CH methylation level calculations, a weighted methylation level metric was utilized, calculated as defined by Schultz *et al*.^60^. In this calculation, a binomial test was employed, with probability of success equal to 0.002 and P-value threshold of 0.001, to predict unmethylated sites. Based on the binomial test, methylation levels of sites predicted to be unmethylated were set to zero. A regional coverage criterion was imposed such that a minimum average read depth of at least 5X per validated cytosine, per strand (10X for combined strands) was required in all samples for a given region to be included. The fraction of cytosines that show a statistically significant amount of methylation in the CH contexts was calculated using methylated and unmethylated reads at each site according to the definition described by Schultz *et al*.^60^. A binomial test was utilized to determine if the observed methylation frequency is above background levels. For this analysis, only sites with validated context and with a minimum depth of at least 5X per strand were included.

### Genomic feature annotation

CpG islands were computationally defined for mm9 using the criteria described by Takai and Jones: length ≥500 bp, GC content ≥55%, ObsCpG/ExpCpG ≥0.65^61^. CpG island shores were defined as regions up to 2 kb flanking either CpG island edge^62,63^. CpG island shelves were defined as regions up to 2 kb flanking CpG island shores^64^. Open sea regions were defined as those greater than 4 kb from the nearest CpG island^65^. Gene features were obtained from RefSeq annotations (February 8, 2016). ChIP-seq peaks for C57BL/6 male adult (8-week) liver were obtained from The Mouse ENCODE Project Consortium (Bing Ren Lab) through Accession Numbers ENCFF001XXU, ENCFF001XXV, ENCFF001XXX, ENCFF001XXY, ENCFF001XXZ, ENCFF001XYA, ENCFF001XYB, ENCFF001YAM, and ENCFF001YAN^41^. For DMR-gene annotation, length-matched random control coordinates were generated using the ‘shuffle’ utility of Bedtools using F > M DMRs (N = 908) with the options ‘-seed 99 -noOverlapping -chrom’ and masking mm9 gap regions. DMR or random null coordinates were overlapped with gene body coordinates, defined as TSS to TES, for all transcripts in our gene model, using the ‘intersect’ utility of Bedtools v2.23.0. DMRs overlapping gene bodies were partitioned into those overlapping TSS (indicated as ‘TSS’) and those not overlapping TSS (indicated as ‘intragenic’). DMRs not overlapping gene bodies are indicated as ‘intergenic’. For assessment of promoter and intragenic methylation levels, TSS and TES coordinates were defined using the most abundant isoform for each gene locus using RNA-seq data. For each RNA-seq sample (N = 20), at each given gene locus, the average normalized FPKM per transcript (N = 35,817) was calculated using Cuffnorm^66^. For each animal, the normalized FPKM of each transcript was calculated such that the sum of FPKM values for each transcript equaled 100 for each gene locus. For each transcript, the average normalized FPKM was calculated across all animals, excluding animals with FPKM of 0 for every transcript for a given locus. Transcripts were ranked by average normalized FPKM for each gene locus, and the most abundant isoform was selected. In the event of a tie, the longest transcript was selected, however if transcripts were the same size, one transcript was randomly selected. Genes were filtered to include only genes greater than 1 kb in length and exclude those annotated as ‘chrN_random’. Top ranking transcripts located on the X chromosome (N = 1,075) and chromosome 3 (N = 1,080) were extracted and utilized for downstream analyses. Promoter regions for each gene (top-ranking transcript) were defined to be a 1 kb region ending at the TSS, and gene body (intragenic) regions were defined to be the region from TSS to TES, as utilized by Schultz *et al*. for XCI calculations^33^.

### Statistical and graphical analyses of DNA methylation data

Statistical and graphical analyses of DNA methylation were conducted using R version 3.4.1^58^. Methylation distribution histogram plots were generated using the ‘multhist’ function of the ‘plotrix’ package using a bin size of 0.1. Boxplots were created using the ‘boxplot’ function of the ‘stats’ package using the default settings. Boxes represent the first to third quartiles of the data distribution and whiskers were drawn to the maximum data value no more than 1.5 times the interquartile distance. P-values for the boxplots were calculated using the Mann-Whitney U-test implemented by the ‘wilcox.test’ of the ‘stats’ package and are two-sided when comparing the methylation level of F > M and M > F DMRs. Heatmaps of weighted methylation levels for CpG islands on chromosomes X and 3 were generated using the ‘heatmap.2’ function of the ‘gplots’ package in R using default parameters for dendogram computation and reordering (based on row or column means), distance (‘dist’, Euclidean method), and hierarchical clustering (‘hclust’, complete method). For CpG island mCG/CG metaplots, CpG islands were filtered to include only islands containing exactly one TSS. CpG islands were oriented to the direction of transcription and length was normalized such that the distance to the upstream and downstream edges were independently scaled relative to the TSS. The length from each CpG island edge to the TSS was divided into 25 variably sized bins. An additional 3 kb to either side of each CpG island (unscaled) was divided into 100 fixed size bins. For each bin of each CpG island, site-specific mCG/CG values were averaged, and then the overall average of each bin across all CpG islands was plotted for males and females. Vertical dashed lines were drawn at the TSS and at each CpG island edge. Smoothed density scatterplots were generated using the ‘smoothScatter’ function of the ‘graphics’ package, which produces a smoothed color density representation of the scatterplot, obtained through a kernel density estimate. Density color keys were generated using the ‘image.plot’ and ‘tim.colors’ functions of the ‘fields’ package.

### XCI gene classification

Assessment of the relationship between female-specific methylation (mCH/CH, mCG/CG) levels and escape from XCI was based on the methodologies described by Ecker and colleagues^32,33^. Two published reports that surveyed allele-specific gene expression of X-linked genes in mouse tissues and cells with skewed XCI were utilized to classify genes subject to or escaping XCI^42,43^. Yang *et al*. used the Patski cell line that contains one Xa from *Mus spretus* and one Xi with an *Hprt* mutation from *Mus musculus* (C57BL/6 J)^43,67^. Additionally, Yang *et al*. performed ChIP-chip analyses to assess H3K27me3 levels in male and female adult liver from B6 mice and demonstrated that the repressive mark is depleted at escaper genes^43^. For this study, we used a list resulting from the intersection of genes escaping XCI in Patski cells and genes depleted of H3K27me3 in both male and female liver (n = 7). Berletch *et al*. assessed brain, spleen, and ovary tissues using a mouse model in which F1 animals contain one Xa that carries a deletion of the *Xist* proximal A-repeat (*Xist*Δ) from *Mus musculus* (C57BL/6 J) and one Xi from *Mus spretus*^42^. Additionally, Berletch *et al*. assessed allele-specific expression in the Patski cell line. For this study, inactivated X-linked genes (n = 177) were defined as the intersection of genes subject to XCI in brain tissue (n = 400) and Patski cells (n = 203). Genes reported to escape XCI were categorized based on level of variability between mouse cell types, including cell-specific escape in brain (n = 6) and Patski cells (n = 52), and common escapers in 2 of 4 (n = 9), 3 of 4 (n = 3), and 4 of 4 (n = 6) cell types. X-inactivation statuses for all genes in B6 and C3 mouse liver were predicted using intragenic mCH/CH and promoter mCG/CG levels. To create training sets, escaper genes were defined as those escaping in at least 3 of 4 tissues examined by Berletch *et al*.^42^ or escaping in Patski cells and depleted of H3K27me3 in both male and female liver in the Yang *et al*. study^43^. For each strain, a linear discriminant analysis (LDA) model was created with female and male intragenic mCH and promoter mCG levels using the ‘lda’ function of the ‘MASS’ package in R (v7.3–47)^68^ with default parameters, and XCI status predictions were made using the ‘predict’ function of the ‘stats’ package in R^69^. Genes with no evidence of expression in B6 or C3 liver (zero total RNA-seq reads in all samples) were excluded. XCI status classifications are listed in Table 2 and Supplementary Table S11.

### Data availability

The data discussed in this publication have been deposited in NCBI’s Gene Expression Omnibus^70^ and are accessible through GEO Series accession numbers GSE106208 and GSE106379.

## Electronic supplementary material

Supplementary Information

Table S1

Table S2

Table S3

Table S4

Table S5

Table S6

Table S7

Table S8

Table S9

Table S10

Table S11

Table S12
