# Supplementary material for: Dosage compensation and DNA methylation landscape of the X chromosome in mouse liver
Source: Sci Rep. 2018 Jul 4;8:10138. doi: 10.1038/s41598-018-28356-3 (PMC6031675; doi:10.1038/s41598-018-28356-3)
Supplement: Supplementary file 1 — Supplementary Information [file 41598_2018_28356_MOESM1_ESM.pdf]

**Supplementary Information:**

# Dosage compensation and DNA methylation landscape of the X chromosome in mouse liver

**Christopher G. Duncan[1], Sara A. Grimm[2], Daniel L. Morgan[3], Pierre R. Bushel[4], Brian D. Bennett[2], NISC Comparative Sequencing Program[5], John D. Roberts[1], Frederick L. Tyson[6], B. Alex Merrick[3], and Paul A. Wade[1,*]**

[1] Epigenetics and Stem Cell Biology Laboratory, National Institute of Environmental Health Sciences, Research Triangle Park, NC, USA.

[2] Integrative Bioinformatics, National Institute of Environmental Health Sciences, Research Triangle Park, NC, USA.

[3] Division of the National Toxicology Program, National Institute of Environmental Health Sciences, Research Triangle Park, NC, USA.

[4] Biostatistics and Computational Biology Branch, National Institute of Environmental Health Sciences, Research Triangle Park, NC, USA.

[5] NIH Intramural Sequencing Center, National Human Genome Research Institute, Rockville, MD, USA.

[6] Division of Extramural Research and Training, National Institute of Environmental Health Sciences, Research Triangle Park, NC, USA.

[*] Corresponding author (email: wadep2@niehs.nih.gov).

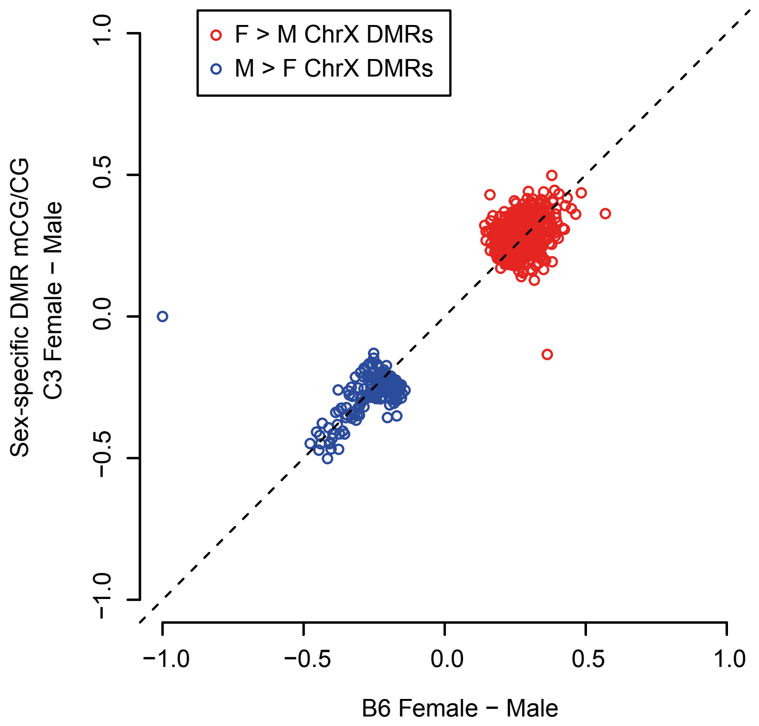


**Supplementary Figure S1.** Strain comparison of female to male methylation differences at sex-specific DMRs on the X chromosome. Scatterplot comparing sex differences (female – male) in sex-specific DMR mCG/CG in the C3 strain (y-axis) versus the B6 strain (x-axis).

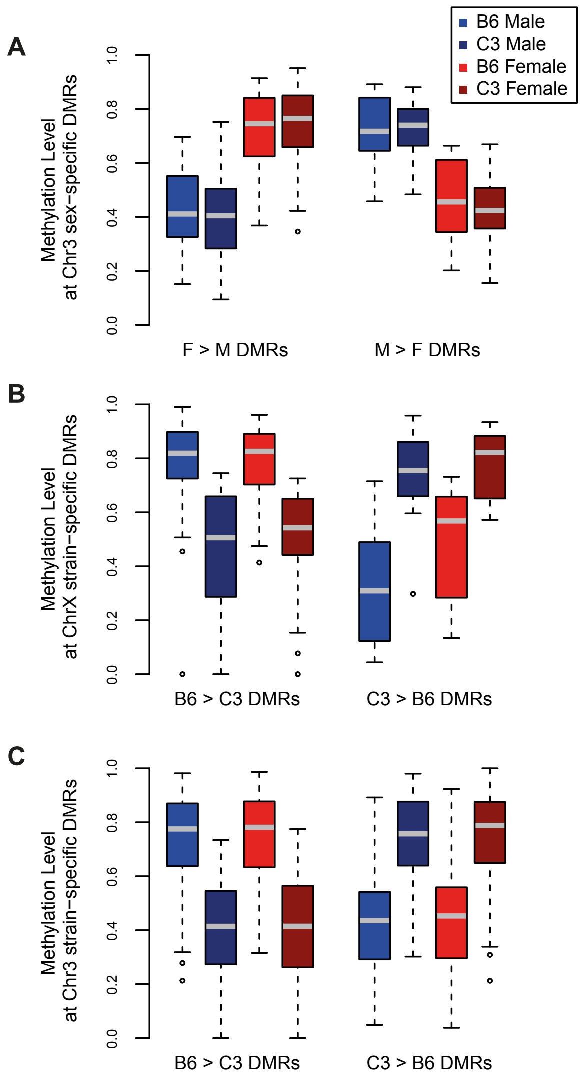


**Supplementary Figure S2.** Methylation levels at strain-specific DMRs and sex-specific autosomal DMRs. (**A**) Boxplots of CG weighted methylation levels at sex-specific DMRs (using DSS) on chromosome 3 for B6 male, C3 male, B6 female, and C3 female mice. (**B**) Boxplots of CG weighted methylation levels at strain-specific DMRs (using DSS) on the X chromosome. (**C**) Boxplots of CG weighted methylation levels at strain-specific DMRs (using DSS) on chromosome 3.

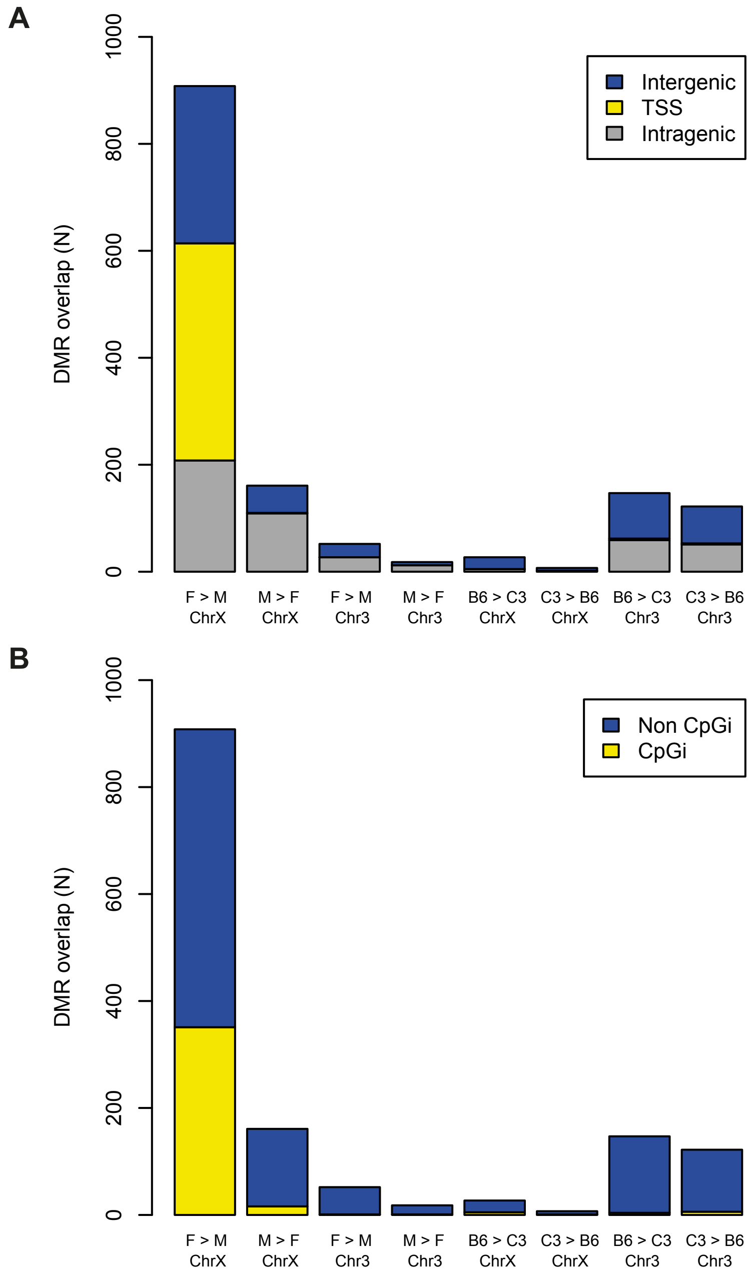


**Supplementary Figure S3.** Sex- and strain-specific DMRs overlapping gene regions and CpG islands. (**A**) Stacked bar plots represent absolute number of DMRs that overlap intergenic regions, TSS, or intragenic (non-TSS) regions for each indicated DMR set. (**B**) Stacked bar plots represent absolute number of DMRs that overlap CpG island regions or non-CpG island regions for each indicated DMR set.

**A**

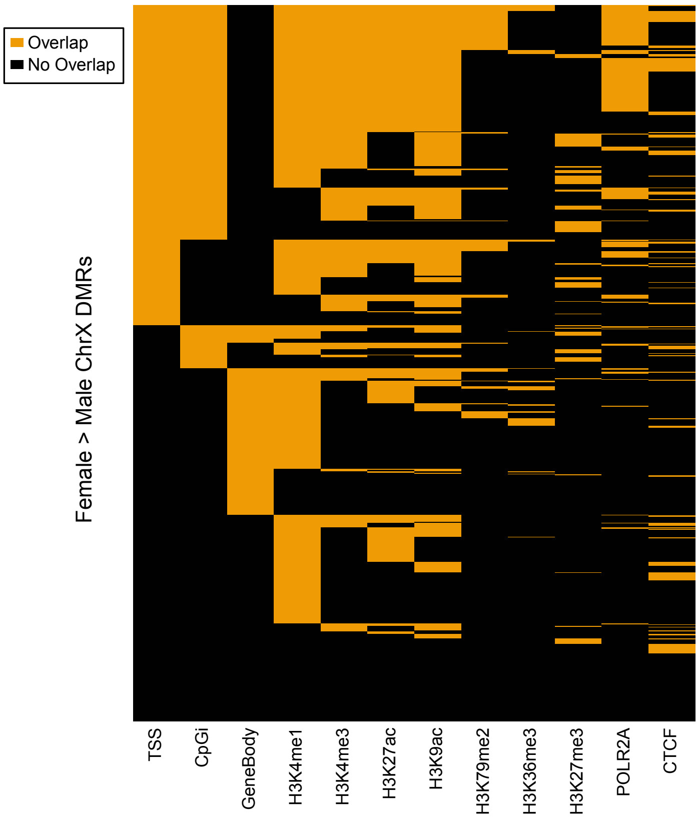


**B**

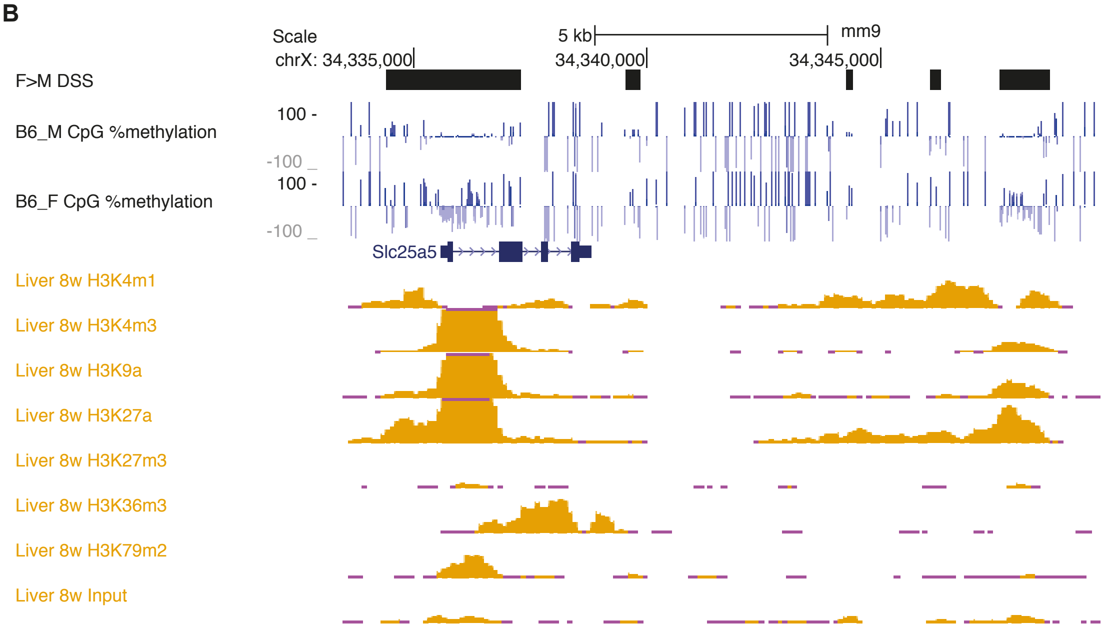


**Supplementary Figure S4.** Female > Male DMRs frequently overlap with histone modification peaks in male liver. (**A**) Heatmap depicting overlap of F > M DMRs (rows) with genomic feature annotations (TSSs, CpG islands, and gene body regions) and ChIP-seq peaks (H3K4me1,

H3K4me3, H3K27ac, H3K9ac, H3K79me2, H3K36me3, H3K27me3, POLR2A, and CTCF) in adult male B6 liver (columns). (**B**) Genome browser view of DNA methylation and ChIP-seq data for the *Slc25a5* locus. Black bars depict F > M DMRs (DSS) occurring at TSS and non-TSS regions. CpG methylation data for B6 males and females, with bars depicting the methylation level at individual validated cytosine positions on the forward (upward bars) and reverse (downward bars) strands. Lower tracks depict histone modification by ChIP-seq signal from ENCODE/LICR.

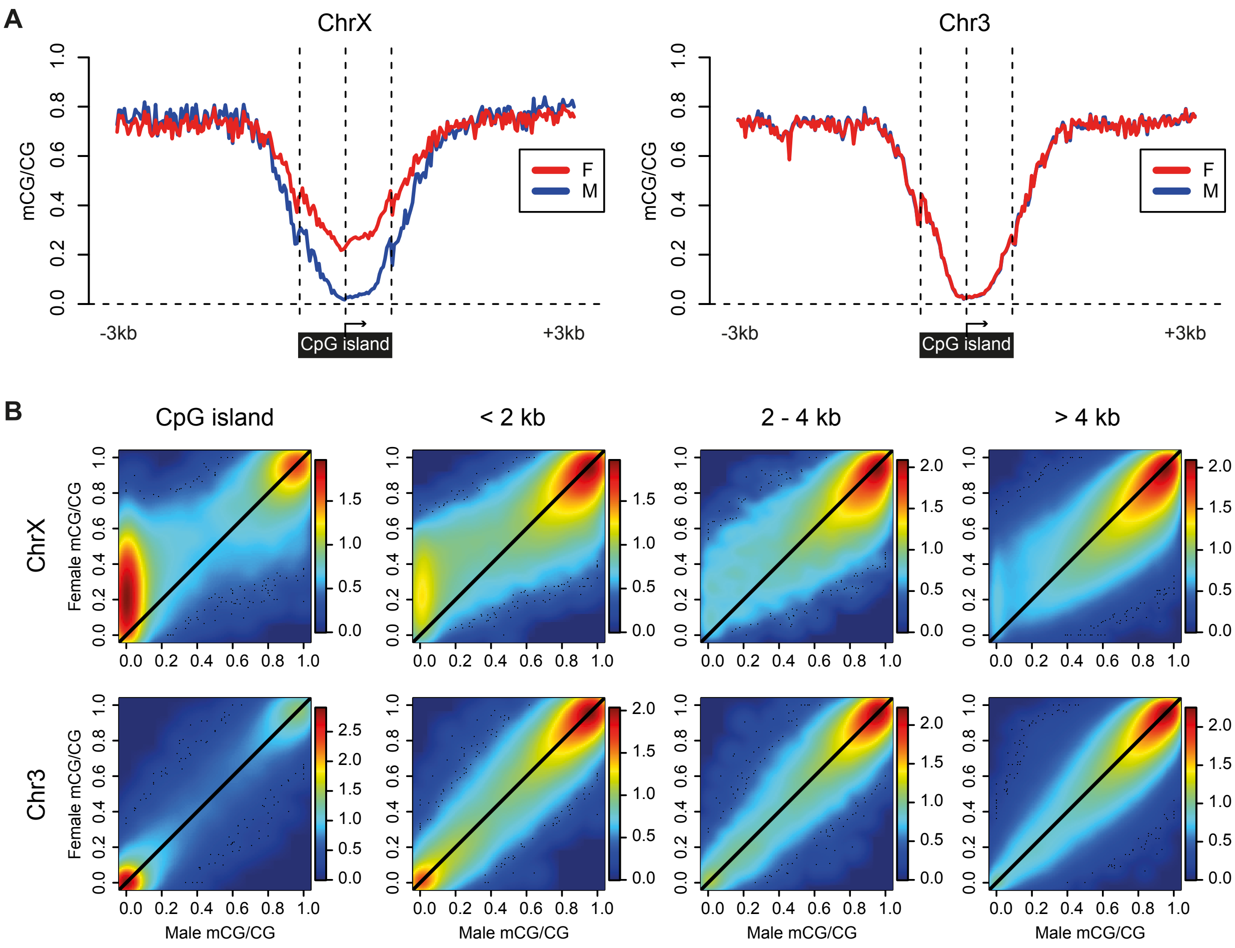


**Supplementary Figure S5.** Evaluation of sexually dimorphic methylation differences on the X chromosome beyond CpG island boundaries using B6 animals. The corresponding analyses using C3 animals are shown in Fig. 4. (**A**) Metaplots of CpG methylation level at CpG islands overlapping exactly one TSS on chromosome X (n = 275) and chromosome 3 (n = 429) for B6 animals (Red, female; Blue, male). CpG islands are oriented to the direction of transcription of the overlapped TSS, and the upstream and downstream CpG island edges are independently scaled relative to the TSS.  An additional 3kb to either side of the CpG island (unscaled) is included. (**B**) Smoothed kernel density scatterplots comparing male and female single-site methylation levels on chromosome X and 3, by proximity to CpG islands. B6 female mCG/CG (y-axis) versus B6 male mCG/CG (x-axis) is plotted for CpG sites with depth of at least 10X in

each sample located in CpG islands, within 0-2 kb from a CpG island edge, within 2-4 kb from a CpG island edge, or greater than 4 kb from the nearest CpG island. Points to the upper-left of the diagonal reflect CpGs with greater female methylation and points to the lower-right of the diagonal reflect CpGs with greater male methylation. Number of chromosome X CpGs included in (C): CpG island, 40,510; < 2 kb, 24,975; 2 – 4 kb, 18,567; > 4 kb, 690,584. Number of chromosome 3 CpGs included in (D): CpG island, 59,276; < 2 kb, 32,974; 2 – 4 kb, 28,059; > 4 kb, 918,690. Density color keys are shown to the right of each plot.

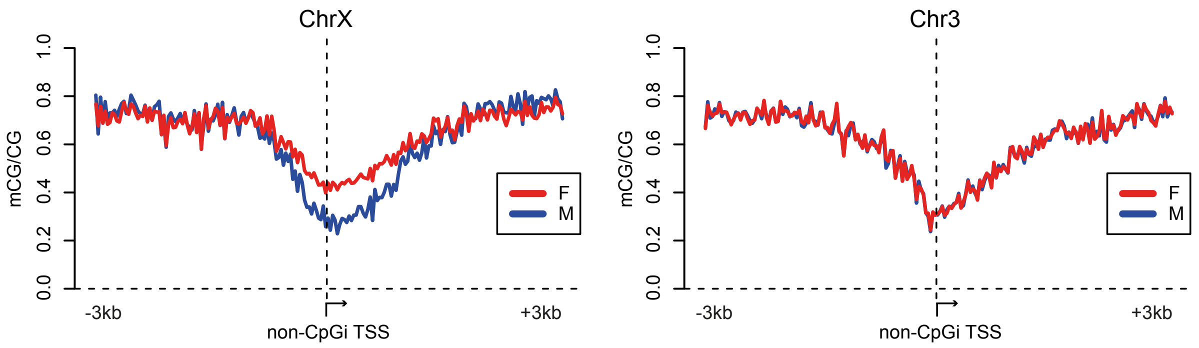


**Supplementary Figure S6.** Evaluation of sexually dimorphic methylation differences on the X chromosome at non-CpG island TSSs. The corresponding analyses using CpG island TSSs are shown in Fig. 4. Metaplots of CpG methylation level at TSSs not located in CpG islands that show evidence of expression (average TPM > 0 in both Male and Female) on chromosome X (n = 256) and chromosome 3 (n = 292) for C3 animals (Red, female; Blue, male). TSSs are oriented to the direction of transcription, and an additional 3kb to either side of the TSS (unscaled) is included.

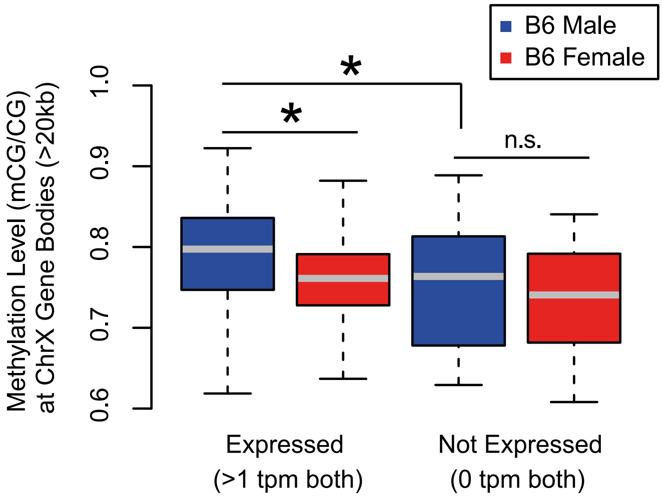


**Supplementary Figure S7.** Relationship between transcription levels and methylation levels at gene body regions. Boxplots of CG weighted methylation levels at gene body regions of expressed genes (average TPM greater than 1 in both male and female mice, N = 166) and genes that are not expressed (average TPM equal to 0 in both male and female mice, N = 72) on chromosome X for B6 male (blue), and B6 female (red) mice. For this analysis, only gene bodies greater than 20 kb in length were included. Genes determined to be not expressed, but that overlapped an expressed gene were excluded. Asterisk (*) indicates $p < 0.01$, Mann-Whitney U-test.

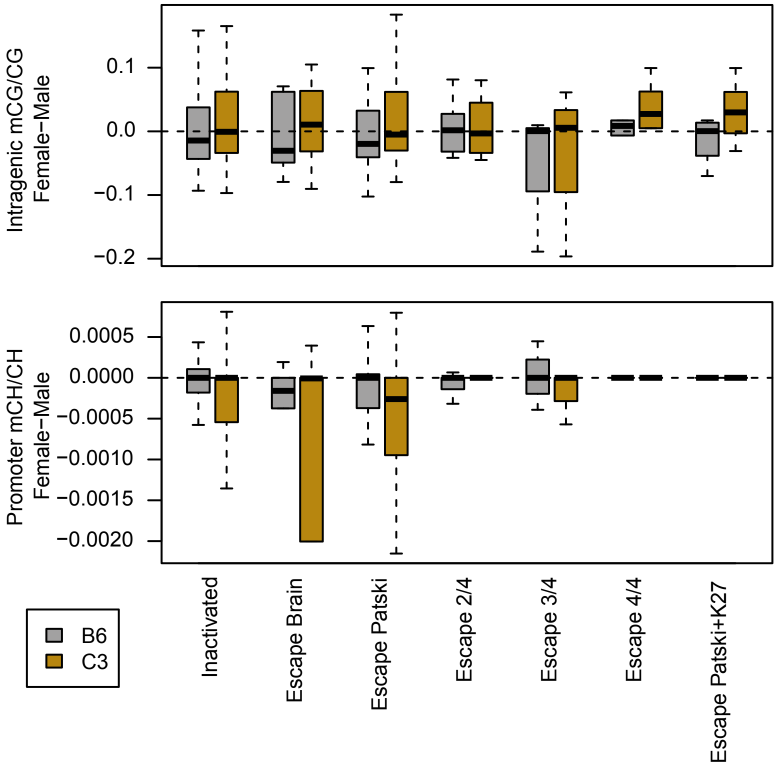


**Supplementary Figure S8.** Sex differences in intragenic mCG/CG and promoter mCH/CH do not effectively distinguish escaper genes from inactivated genes. The corresponding analyses using intragenic mCH/CH and promoter mCG/CG for the same gene sets are shown in Fig. 5B. Boxplots of sex differences (female – male) in intragenic mCG/CG and promoter mCH/CH at genes with previously demonstrated XCI status in multiple mouse cell types. Classifications include genes subject to XCI in both brain and Patski cells (n = 177), brain-specific escapers (n = 6), Patski-specific escapers (n = 52), genes escaping in 2 of 4 (n = 9), 3 of 4 (n = 3), or 4 of 4 (n = 6) cell types analyzed by Berletch et al. [42], or genes escaping in the Patski cell line and depleted of H3K27me3 in adult mouse liver (n = 7), as demonstrated by Yang et al. [43].

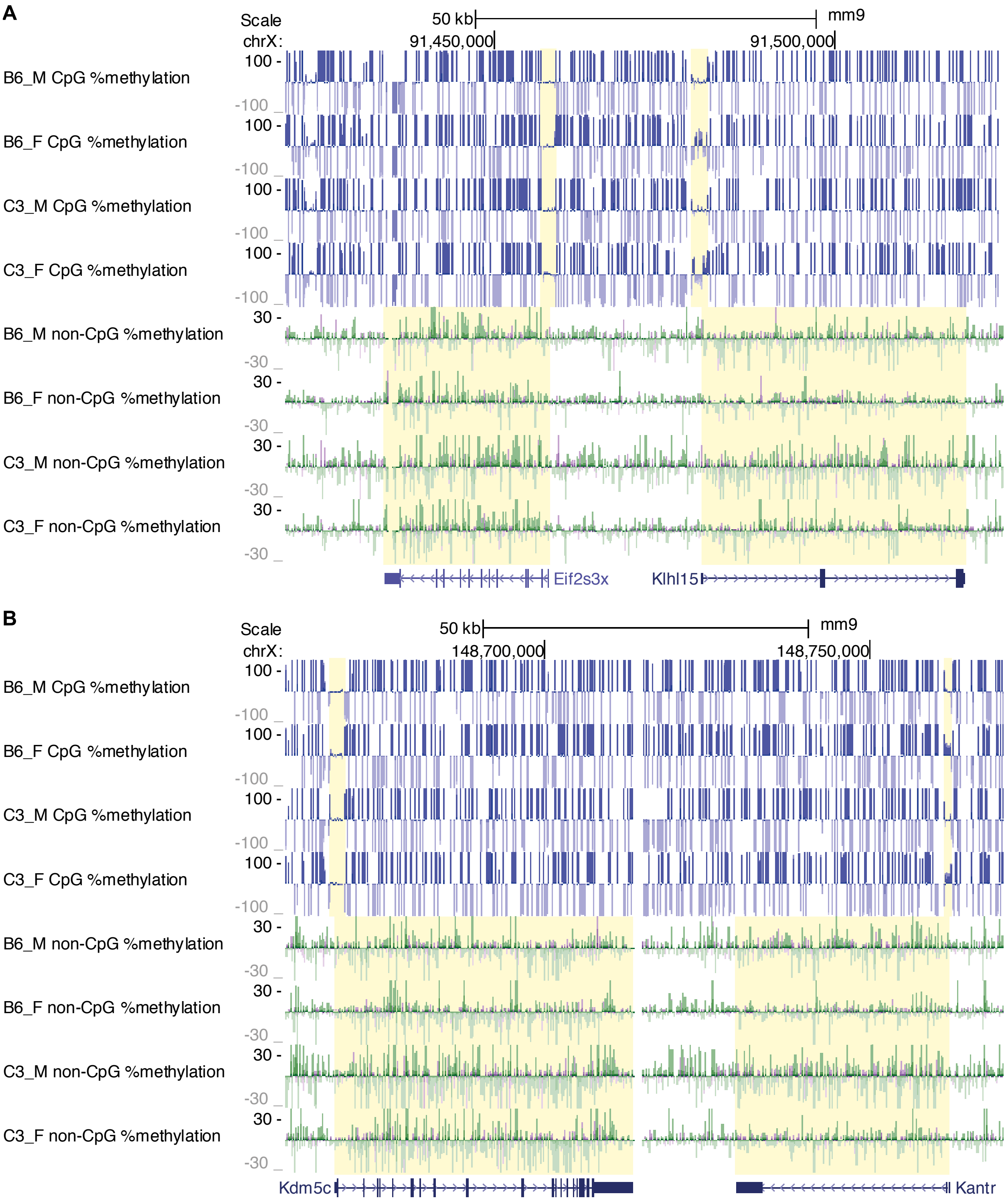


**Supplementary Figure S9.** Genome browser examples of escape versus non-escape genes in B6 and C3 mouse liver. Genome browser views of DNA methylation data for the (A) *Eif2s3x* locus and the (B) *Kdm5c* locus. Bars depict the methylation level at individual validated cytosine

positions (CG, blue; CH, green) on the forward (upward bars) and reverse (downward bars) strands. For comparison, each panel includes an escape gene (*Eif2s3x* in A; *Kdm5c* in B) and a neighboring gene that is subject to XCI (*Klhl15* in A; *Kantr* in B). Regions useful in distinguishing escape genes from genes subject to XCI (promoter mCG, intragenic mCH) are highlighted with yellow boxes. When comparing males and females within each strain (B6, C3), genes subject to XCI (*Klhl15*, *Kantr*) exhibit sex-specific promoter mCG (upper tracks, CpG %methylation), as promoter CG sites are largely unmethylated in males and partially methylated in females. Genes subject to XCI also exhibit sex-specific intragenic mCH (lower tracks, non-CpG %methylation) as gene body CH sites are more highly methylated in males than in females. However, for genes escaping XCI (*Eif2s3x*, *Kdm5c*), promoters do not exhibit sex-specific mCG, as promoter CG sites are largely unmethylated in both males and females. Further, in genes that escape XCI, mCH is enriched in female gene bodies, resulting in similar intragenic mCH levels in males and females.

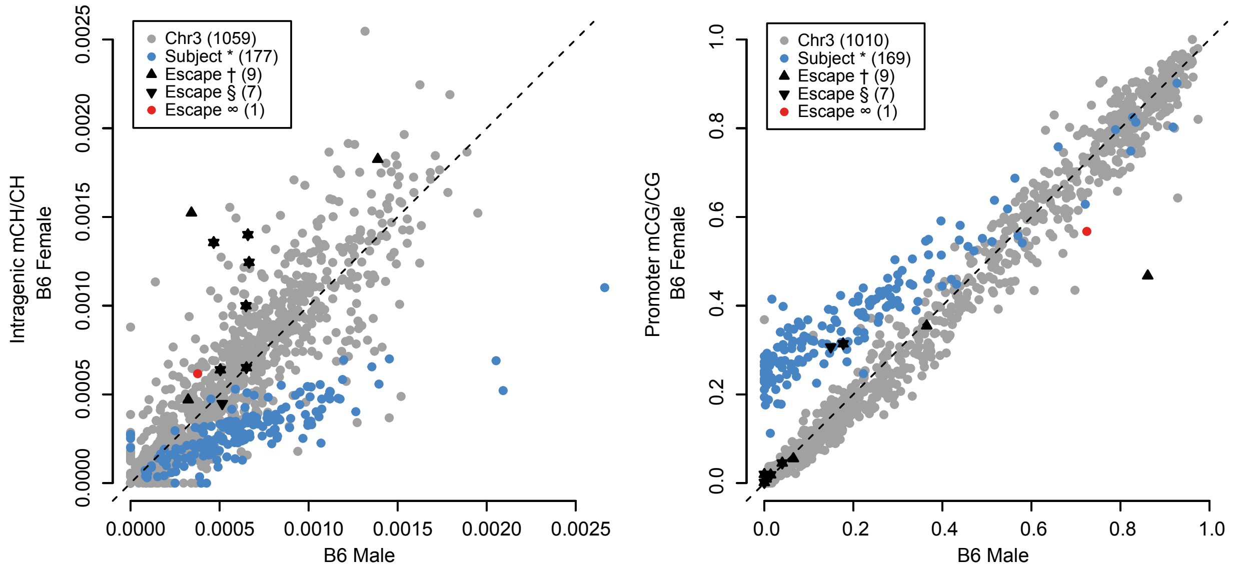


**Supplementary Figure S10.** Evaluation of non-CpG methylation enrichment in genes escaping XCI in mouse liver using B6 animals. The corresponding analyses using C3 animals are shown in Fig. 5C. Gene-level scatterplots comparing B6 male and B6 female intragenic mCH/CH and promoter mCG/CG for mouse genes by XCI status. Mouse genes reported as inactivated or escapers: Berletch et al. subject to XCI in both brain and Patski cells (*), Berletch et al. escape in at least 3 of 4 cell types ( † ), Yang et al. escape in Patski cells and depleted of H3K27me3 in adult liver ( § ). Predicted escaper genes (∞) and autosomal genes (Chr3) are also indicated.

## Supplementary Tables

**Supplementary Table S1.** Summary of RNA-seq read pair filtering and mapping.

**Supplementary Table S2.** Pairwise differential expression comparisons.

**Supplementary Table S3.** Comparison of sex-biased genes across strains.

**Supplementary Table S4.** Comparison of sex- and strain-biased gene expression.

**Supplementary Table S5.** Functional annotation of sex-biased autosomal genes.

**Supplementary Table S6.** Summary of validated cytosines.

**Supplementary Table S7.** Summary of WGBS depth normalization.

**Supplementary Table S8.** Summary of CG coverage.

**Supplementary Table S9.** Differentially methylated regions.

**Supplementary Table S10.** Weighted methylation levels for promoter and gene body regions on chromosomes X and 3.

**Supplementary Table S11:** Classification of XCI status.

**Supplementary Table S12.** Bisulfite conversion rates as assessed by read pairs mapped to Enterobacteria phage λ.
